# Editorial: Harnessing machine learning for enhanced biomedical diagnosis and early disease detection: bridging data science and healthcare

**DOI:** 10.3389/fonc.2026.1895923

**Published:** 2026-06-22

**Authors:** Mahendra Gawali, Hsiang-Chen Wang, Arvind Mukundan

**Affiliations:** 1 Department of Computer Science Engineering, School of Engineering and Technology, Sanjivani University, Singnapur, India; 2 Department of Mechanical Engineering, National Chung Cheng University, Min Hsiung, Chia-Yi, Taiwan; 3 Department of Medical Research, Dalin Tzu Chi Hospital, Buddhist Tzu Chi Medical Foundation, Dalin, Chiayi, Taiwan; 4 Department of Biomedical Imaging, Chennai Institute of Technology, Sarathy Nagar, Chennai, India

**Keywords:** cancer detection, computer-aided diagnosis, deep learning, healthcare, machine learning

The swift progression of Machine Learning (ML) and Deep Learning (DL) technology has transformed healthcare, especially in disease prediction and diagnosis (1). These developments have resulted in enhanced cancer detection rates, facilitating prompt interventions and tailored treatment strategies (2). The integration of AI and ML in biomedical engineering fosters innovation and enhances decision-making, enabling advancements in healthcare technologies. This will enable us to find potential state-of-the-art solutions by employing new frameworks for applications such as medical imaging, wearables, and biomanufacturing (3). This Research Topic, “Harnessing Machine Learning for Enhanced Biomedical Diagnosis and Early Disease Detection: Bridging Data Science and Healthcare,” was established to compile innovative research and thorough reviews aimed at addressing the critical challenge of timely and accurate cancer detection, essential for improving patient outcomes.

Liu et al. created a classification algorithm using 4,806 endoscopic ultrasonography images from 219 patients. DL and radiomic features are derived using t-tests and the LASSO method, utilizing a univariate model. The model attained an AUC of 0.95, with sensitivities, specificities, positive predictive values, negative predictive values, and accuracies of 100.00%, 82.86%, 60.00%, 100.00%, and 86.36%, respectively.

Xiao et al. developed a ML model to categorize diabetic microvascular complications, employing data obtained from clinical and laboratory sources involving 1,498 patients. This is classified into: diabetes without complications and with microvascular complications. Nine classification models were assessed, demonstrating that the Gradient Boosting Decision Tree (GBDT) model had superior performance. The risk factors for diabetic microvascular complications encompass urea, fibrinogen (FIB), prothrombin time (PT), D-dimer (DD), creatine kinase MB isoenzyme (CKMB), lipoprotein(a), activated partial thromboplastin time (APTT), triglycerides (TG), and cholinesterase (CHE).

Li et al. analyzed a total of 197 surgically resected lung cancer patients, dividing them into a training and testing cohort of 137 and 60 respectively. Clinical data, spectral dual-layer detector CT (SDCT) images, and tumor tissue specimens for histological diagnosis of Spread Through Air Spaces (STAS) were employed. To attain enhanced results, radiomics, DL, and DL radiomics models are utilized for analysis and the prediction of nomograms. Logistic Regression (LR) had superior performance demonstrating enhanced predictive skills attaining the best accuracy and AUC of 0.918.

Li et al. developed a noninvasive radiomics-based ML model, incorporating clinicopathological characteristics, to predict instability status in right colon cancer, functioning as a preoperative decision-making instrument for clinical practice. 247 patients were assessed using patient tumor data and CT scan findings. CT images were analyzed using 3D Slicer to identify regions of interest (ROI) for model training and enhancement of image-based analysis. Random forest (RF) algorithm demonstrated greater performance compared to the various techniques assessed, with AUC values of 0.99 in the training set and 0.97 in the test set.

Fully-field digital mammography (FFDM) is the conventional technique for breast cancer screening (BCS). Digital breast tomosynthesis (DBT) enhances cancer detection and reduces unnecessary biopsies relative to full-field digital mammography (FFDM). To address this issue, Beaumont et al. examined Computer-Aided Diagnosis (CAD) as a technique for BCS. In the evaluation of a breast cancer cohort of 1,816 individuals who had both FFDM and DBT at two Hologic facilities, several cases displayed conflicting AI ratings. AI-driven and lossless models shown effectiveness in classifications for 82.2% of perpendicular scoring (PS) of masses and 67.3% for calcifications. When evaluated by CAD, FFDM and DBT provided insights, albeit inconsistent diagnoses were inevitably present. The CAD model’s efficacy in reclassifying conflicting diagnoses has enhanced.

Preoperatively predict tumor deposits (TDs) in patients with advanced cancer (AGC) utilizing intratumoral and peritumoral radiomics derived from CT scans. To address this issue, Yao et al. examined a total of 374 patients from two medical institutes, categorizing them into training (186), validation (80), and test (108) cohorts. Created using intratumoral and peritumoral radiomics models derived from three-dimensional ROIs. The combined intratumoral-peritumoral radiomics model had a superior AUC across all cohorts (0.925, 0.865, 0.878 for training, validation, and test cohorts, respectively). Intratumoral and peritumoral radiomics offer substantial insights into tumor dynamics, while the CT-based ensemble model can preoperatively predict tumor dynamics in patients with advanced gastric cancer.

Ma et al. states that incorporation of AI into clinical practice is hindered by inadequate clinical validation for generative AI in image enhancement and a lack of multi-center efficacy data for commercial CAD systems. This study analyzes recent advancements in technology assessment, challenges in clinical translation, and prevalent methodological limitations, such as restricted sample sizes and retrospective single-center designs that often lead to an overestimation of actual AI performance in real-world contexts.

Huo et al. constructed a nomogram model utilizing traditional Chinese Medicine (TCM) pulse mapping characteristics to forecast the probability of diabetic foot (DF) in individuals with type 2 diabetes mellitus (T2DM). Patients were classified into DF (242) and T2DM (243) according to the existence of diabetic foot (DF). Calibration decision curve analysis (DCA) was utilized to improve the model’s performance. The nomogram model achieved an AUC of 0.786 and an accuracy of 0.7113, enabling the prediction of diabetic foot (DF) risk in patients with T2DM and offering TCM-based reference markers for early DF risk assessment.

Song et al. investigated the genetic predisposition linked to MTHFRC677T and its interaction with modifiable risk variables in patients with diabetic nephropathy in northern China, and developed a risk prediction model for early diagnosis. A total of 397 patients with type 2 diabetes were recruited from the Second Hospital of Shanxi Medical University, classified into diabetic nephropathy (DN) and non-diabetic nephropathy (N-DN). A LR model employed to identify independent risk factors. Eight variables, including blood urea nitrogen (BUN) and urinary albumin-to-creatinine ratio (ACR), exhibited significant correlations with DN, with odds ratios (ORs) ranging from 1.003 to 4.572. The resulting nomogram demonstrated notable predictive accuracy and discrimination, assisting in the diagnosis of diabetes risk and enabling the development of management and treatment strategies for diabetic nephropathy.

Yang et al. evaluated the effectiveness of the triglyceride–high-density lipoprotein glucose–body (TyHGB) index in detecting diabetic kidney disease (DKD) in persons with type 2 diabetes mellitus (T2DM). The LR analysis demonstrated an independent and non-linear association between the TyHGB score and DKD. Among the subjects, 286 (20.7%) were diagnosed with DKD. After adjusting for demographic, clinical, and biochemical factors, the TyHGB score maintained a significant correlation with DKD (OR = 1.11, 95% CI: 1.05–1.17, p < 0.001). The TyHGB model improved prediction performance with an AUC of 0.715 and provided notable reclassification benefits. Compared to the TyG index, TyHGB shown superior effectiveness for the early identification and risk assessment of diabetic kidney disease in clinical environments.

Coronary artery disease demonstrates a strong bidirectional relationship with diabetes mellitus. Wang et al. assess a ML-based predictive model for coronary artery disease patients with diabetes comorbidity, employing data from 389 individuals. The dataset is divided into training (273) and internal validation (116) sections. LASSO regression is utilized, succeeded by backward stepwise Cox regression analysis, while the RF algorithm corroborates the clinical significance of the chosen variables. The integrated model demonstrated strong performance, with an AUC of 0.838 for training and 0.824 for validation at intervals of 3, 5, and 8 years. The model exhibits exceptional performance by employing nine parameters, showcasing strong predictive powers, while its nomogram feature enables customized risk assessment for clinical decision-making.

Follicular thyroid carcinoma (FTC) is the second most common variant of thyroid cancer. Yuan et al. developed a ML approach employing radiomic features to improve the risk stratification of FTC. A total of 277 people with verified follicular thyroid neoplasms (163 follicular adenomas, 63 follicular tumors with indeterminate malignant potential, and 51 follicular thyroid carcinomas) were included. Intratumoral and peritumoral areas were delineated by preoperative ultrasound imaging. Three ML models: LR, RF, and Support Vector Machine (SVM). The RF-based clinical ultrasound model achieved the highest accuracy. Among the clinical ultrasonography models, the LR model, which employs peritumoral radiomics, exhibited the highest accuracy in testing compared to the model that integrates both intratumoral and peritumoral radiomics.

Diabetic foot ulcer (DFU) reduces quality of life and increases mortality risk. Zhang et al. aimed to reduce the risk of diabetic foot ulcers in diabetic patients by a ML-based approach. The study complied with the TRIPOD+AI standards, employing NHANES data. The model was trained with LR, K-NN, RF, XGBoost, and SVM classifiers, which were evaluated against a dataset obtained from the Second Affiliated Hospital of Zunyi Medical University for validation purposes. A total of 1,857 participants from NHANES and 807 individuals recruited for the testing dataset. The RF model demonstrates the highest AUC of 0.81 for NHANES, whereas the other models produce acceptable outcomes.

Human brucellosis (HB) presents a considerable clinical challenge, leading to extended disability. To address this issue, Wang et al. developed a web-based predictive tool to evaluate ML models for forecasting chronic progression utilizing clinical and laboratory data from 555 patients diagnosed with brucellosis. Boruta and recursive feature removal were performed using six ML models. Among the patients, 144 (25.9%) manifested persistent brucellosis. The RF exhibits the highest AUC (0.782) and exceptional calibration; SHAP analysis indicated TG, HDL-C, UA, eosinophil count, PA, ALT, BUN, and GLB as key predictors.

Gestational diabetes mellitus (GDM) is a prevalent metabolic disorder linked to pregnancy. To tackle this issue, Ji et al. developed a preliminary prediction model for gestational diabetes mellitus by incorporating first-trimester metabolomic profiles with clinical risk variables. They identified 528 unique metabolites and 20 associated metabolic pathways. LASSO regression found 11 distinct metabolites and 3 clinical features for the development of ML models. Among the six ML models, the multilayer perceptron demonstrated the highest performance, achieving an AUC of 0.984 in validation. SHAP analysis finds GlcCer (d18:1/16:0) and triglycerides as significant predictors of GDM.

Wang et al. forecasted postoperative hyperglycemia (POH) utilizing ML models, including 393 non-diabetic patients who underwent radical gastrectomy for gastric cancer. Thirty-eight preoperative clinical parameters were collected. POH, characterized by a plasma glucose concentration over 7.8 mmol/L within 24 hours following surgery, was noted in 42.7% of patients. Nine ML models were developed and assessed, with the SVM-radial model exhibiting the highest performance, attaining an AUC of 0.758, an accuracy of 0.724, an F1-score of 0.743, and a recall of 0.750. Key predictors included operative duration, dietary risk assessment, and alkaline phosphatase concentrations. SHAP research confirmed their contribution, endorsing practical applicability for early-stage risk classification and individualized glycemic management in surgical settings.

Lupus nephritis (LN), a severe complication of systemic lupus erythematosus (SLE), presents ongoing challenges in its prediction and therapy owing to its erratic course. Garcia-Bañol et al. provides a review that consolidates current technologies for employing ML models in the prediction, diagnosis, and monitoring of lymph nodes, including diagnostic procedures. ML methodologies applied to multimodal data sources like as clinical, laboratory, imaging, histological, and omics data improve clinical decision-making, laboratory analysis, imaging, histopathology, and omics.

Yang et al. developed a model that may preoperatively predict the pathological classification of Retroperitoneal liposarcoma (RLPS), hence enhancing treatment strategies. This retrospective investigation of 114 patients with RLPS investigated the capacity of radiomic characteristics derived from plain CT scans to predict pathological subtypes prior to surgery. Features were chosen by LASSO regression, yielding a radiomic signature and nomogram for the diagnosis of dedifferentiated liposarcoma (DDLPS). Increased Ki-67 expression and indistinct tumor margins were independent prognostic indicators for DDLPS. The nomogram demonstrated an AUC of 95% for the training set and 0.89% for the validation set, providing a greater net clinical benefit than the radiologist.
